# Identification of Potential Sites for Tryptophan Oxidation in Recombinant Antibodies Using *tert*-Butylhydroperoxide and Quantitative LC-MS

**DOI:** 10.1371/journal.pone.0017708

**Published:** 2011-03-03

**Authors:** Miriam Hensel, Rebecca Steurer, Juergen Fichtl, Carsten Elger, Frank Wedekind, Andreas Petzold, Tilman Schlothauer, Michael Molhoj, Dietmar Reusch, Patrick Bulau

**Affiliations:** 1 Pharma Development Penzberg, Roche Diagnostics GmbH, Penzberg, Germany; 2 Pharma Research Penzberg, Roche Diagnostics GmbH, Penzberg, Germany; University of Helsinki, Finland

## Abstract

Amino acid oxidation is known to affect the structure, activity, and rate of degradation of proteins. Methionine oxidation is one of the several chemical degradation pathways for recombinant antibodies. In this study, we have identified for the first time a solvent accessible tryptophan residue (Trp-32) in the complementary-determining region (CDR) of a recombinant IgG1 antibody susceptible to oxidation under real-time storage and elevated temperature conditions. The degree of light chain Trp-32 oxidation was found to be higher than the oxidation level of the conserved heavy chain Met-429 and the heavy chain Met-107 of the recombinant IgG1 antibody HER2, which have already been identified as being solvent accessible and sensitive to chemical oxidation. In order to reduce the time for simultaneous identification and functional evaluation of potential methionine and tryptophan oxidation sites, a test system employing *tert*-butylhydroperoxide (TBHP) and quantitative LC-MS was developed. The optimized oxidizing conditions allowed us to specifically oxidize the solvent accessible methionine and tryptophan residues that displayed significant oxidation in the real-time stability and elevated temperature study. The achieved degree of tryptophan oxidation was adequate to identify the functional consequence of the tryptophan oxidation by binding studies. In summary, the here presented approach of employing TBHP as oxidizing reagent combined with quantitative LC-MS and binding studies greatly facilitates the efficient identification and functional evaluation of methionine and tryptophan oxidation sites in the CDR of recombinant antibodies.

## Introduction

The oxidative degradation of pharmaceutical proteins has been reviewed by several authors [1], [2], [3], [4]. Amino acid residues that are particularly susceptible to oxidation include cysteine, methionine, tryptophan, histidine, and tyrosine, in that order. Since recombinant antibodies do not contain significant amounts of free cysteine, cysteine oxidation will not be addressed here. Instead, we focus on the oxidation of methionine (Met) and tryptophan (Trp). Oxidation of Met residues in the constant domains of recombinant antibodies has been demonstrated to impact the interaction with the neonatal Fc receptor and binding to the Fcγ receptors [5]. Induction of Trp oxidation in the complementary-determining regions (CDRs) of a monoclonal antibody by photooxidation resulted in a progressive loss of target binding and biological activity [6].

Oxidation of Met can be induced by incubation with oxidizing agents like H_2_O_2_ [2], [5], [7], [8] or *tert*-Butylhydroperoxide (TBHP) [3], [5], [6], [9], [10], [11], by ultraviolet light irradiation [6], [12], and is also observed in pharmaceutical antibodies at elevated temperature conditions [10], [12]. Trp residues have been oxidized with oxygen radicals [13] or with Fe(II)/EDTA/Asc [14], and oxidation of Trp residues in monoclonal antibodies using ozone and UV irradiation has been reported recently [6], [15]. Significant Trp oxidation in the parathyroid hormone (PTH) was also reported by Ji et al., who used 2,2′-Azobis(2-amidinopropane) dihydrochloride (AAPH) as stress condition [2]. To our knowledge, none of the reported oxidizing agents have ever been used to induce oxidation of solvent accessible Trp residues in a recombinant antibody. In the present study, an approach employing TBHP as oxidizing agent and tryptic peptide mapping combined with quantitative liquid chromatography-mass spectrometry (LC-MS) for the simultaneous induction, identification and quantification of Trp oxidation in recombinant antibodies was developed. The described test system allowed us to identify a Trp residue in the complementary-determining region that displayed significant oxidation in a real-time stability and elevated temperature study.

## Results

**Figure 1:**
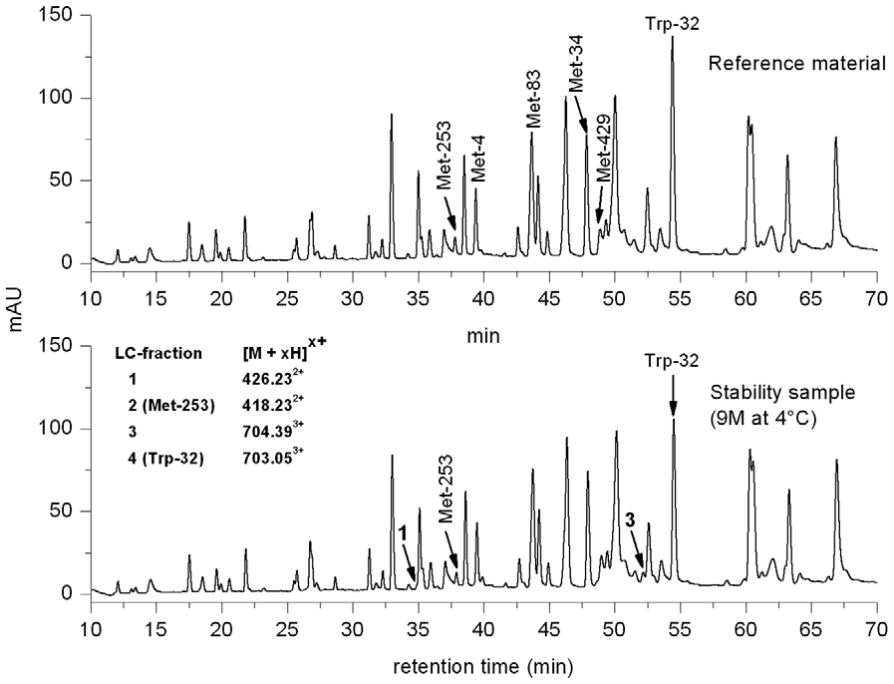
LC-separation UV-profiles of the real-time stability sample (9 months at 4°C) and the corresponding reference material (RS, stored at −70°C) after tryptic cleavage. The *m/z*-values of Met-253 and Trp-32 containing tryptic antibody peptides, obtained by LC-ESI-MS, are listed in the inset along with their corresponding LC fraction number. Peak identification and quantification performed by LC-MS. Chromatographic conditions are described in the materials and methods.

**Figure 2:**
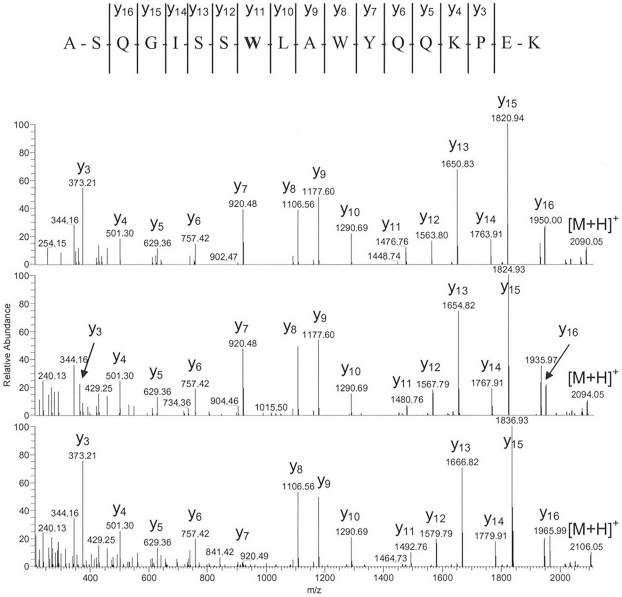
Low-energy CID mass spectra and resulting amino acid sequence of the triply protonated quasi molecular ions of the non-oxidized (upper panel) and oxidized (medium and lower panel) tryptic light chain fragment 25–42 at *m/z* 703.05, 704.39, and 708.38. The oxidized Trp-32 residue is highlighted in bold-face.

**Figure 3:**
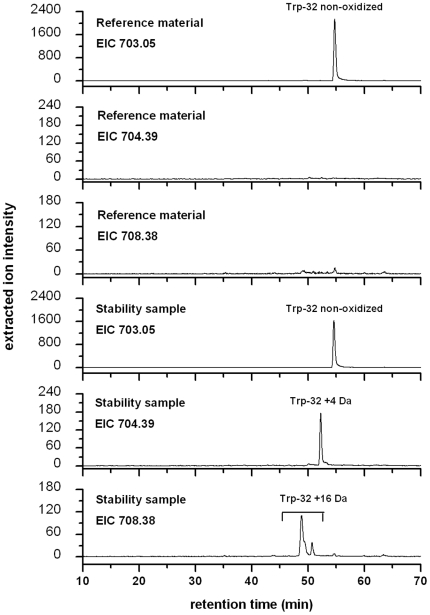
Extracted ion chromatograms (EIC) of *m/z* 703.05, 704.39, and 708.38 from the reference material and the stability sample.

The selected recombinant IgG1 antibody displayed significant oxidation of Trp and Met in a real-time stability study. Figure 1 illustrates the LC-UV profiles of the stability sample (9 months at 4°C) and the corresponding reference material (stored at −70°C) after tryptic cleavage. Significant differences in peak intensities of the tryptic peptides with retention times of 38 min (LC-fraction 2, containing heavy chain Met-253) and 54 min (LC-fraction 4, containing light chain Trp-32) could be observed. No significant differences in peak intensities were observed for the tryptic peptides containing light chain Met-4, heavy chain Met-34, heavy chain Met-83, and heavy chain Met-429 by LC-UV analysis. Moreover, two low abundant unknown peaks were observed in the UV trace of the stability sample at the retention time of 35 min (LC-fraction 1) and 52 min (LC-fraction 3) not observed in the reference material. Oxidized peptides typically elute several minutes prior to the parent peptides, as the resulting products are more hydrophilic. The existence of oxidized Met-253 and Trp-32 was further suggested by LC-MS analysis results, in which mass differences of +16 Da (LC fraction 1 versus 2) and +4 Da (LC fraction 3 versus 4) were observed for the detected peptides (Figure 1, inset). Sequence determination of the oxidized tryptic peptides was carried out on-line by low-energy CID. The resulting MS/MS spectra of peptides containing Trp-32 (summarized in Figure 2) displayed almost complete sequence information for all selected precursor ions (Figure 1, inset). Based on the fragment ions in the MS/MS spectra [16], the tryptic peptide of LC fraction 1 was positively identified as an oxidized variant (Met sulfoxide of Met-253) of the parent peptide in fraction 2. Analysis of the MS/MS spectra of the peptide species in LC fraction 3 resulted in the verification of the Trp-32 derivative kynurenine (Figure 2; medium panel). In addition, the existence of the Trp-32 derivatives 5-hydroxytryptophan, or ox-indole alanine (both with the identical *m/z* of 708.38^3+^) not detected by LC-UV analysis was identified by LC-MS/MS (Figure 2; lower panel) at a retention time of 48 min and 51 min (Figure 3; lower panel). The masses of the C-terminal fragment y series ions, y_11_ to y_16_, of the oxidized peptides are 4 and 16 Da higher than that of the parent peptide (Figure 2; upper panel), respectively. Moreover, the detected fragment ions do not suggest significant oxidation of Trp-35 (detectable at y_8_ to y_10_). For Met-253 and Trp-32 we did not observe mass signals in the total ion current chromatogram (TIC) suggesting the presence of Met sulfone (+32 Da) and N-formyl-kynurenine (+32 Da).

Quantitative differences in peptide abundance of Trp-32 containing peptides were displayed by LC-MS analysis followed by the preparation of extracted ion chromatograms (Figure 3). Two peptide species with identical MS/MS spectra were found for *m/z* 708.38 of the stability sample. The identity of the peptide species (5-hydroxytryptophan versus ox-indole alanine) remains to be elucidated in further studies. The extent of oxidation at each active site was determined by selected ion current chromatogram analysis of the oxidized product and its parent peptide using the quantification software GRAMS/32. The quantitative results of Met-83, Met-253, and Trp-32 oxidation of the stability sample and the reference standard are summarized in Table 1. Beside the significant oxidation at the solvent accessible Met-253 (26%) and trace amounts of oxidation at the buried Met-83 (1.3%), we found that all of the Met residues of the stability sample were slightly oxidized, albeit to different extents (Met-4 (1.4%), Met-34 (2.6%), and Met-429 (5.0%)). These results are in agreement with previous studies on the evaluation of IgG1 conserved Met residues (Met-253 and Met-429) in recombinant antibodies [5], [6], [7], [8], [9], [10], [12], [17], [18]. However, beside the oxidation of Trp-32 no additional light or heavy chain Trp was found to be oxidized at detectable levels. Next, we analyzed the selected antibody after prolonged incubation (3 M) at elevated temperature (35°C) conditions. Again, we found increased levels of Met-253 and Trp-32 when compared with the reference material (Table 1), albeit to different extend when compared with the stability sample (9 months at 4°C). Subsequently, we evaluated if the oxidation of Trp-32 can be demonstrated using chemical agents as a model stress system. Oxidation of exposed Met residues can be induced by incubation with oxidizing agents like H_2_O_2_ [2], [5], [7], [8] or TBHP [3], [5], [6], [9], [10], [11]. For the depiction of method development we focused on the oxidation degree of Met-253 (most susceptible for oxidation), Met-83 (no susceptibility to oxidation in previous studies), and Trp-32.

**Table 1:** Relative quantification of antibody methionine and tryptophan oxidation (Met-83, Met-253, and Trp-32) by ion current chromatogram analysis of the oxidized product and its parent peptide using the quantification software GRAMS/32TM (columns 1–4).

	Met-83 (+16 Da)	Met-253 (+16 Da)	Trp-32 (+4 Da)	Trp-32 (+16 Da)	% Fragment % Monomer % Aggregat	% Target Binding
**Reference material (n = 4)**	0.2 (±0.1)	2.1 (±0.1)	0.5 (±0.1)	0.6 (±0.1)	<0.199.3 (±0.1)0.6 (±0.1)	100 (±10)
**Stability sample (9 M at 4**°**C)**	1.3	25.6	8.3	8.1	0.497.72.0	n.d.
**Stability sample (3 M at 35**°**C)**	0.3	7.6	1.6	3.1	4.894.70.6	n.d.
**Control (24 h; n = 4)**	0.6 (±0.3)	2.3 (±0.4)	0.5 (±0.1)	0.6 (±0.2)	0.7 (±0.1)98.7 (±0.1)0.5 (±0.1)	100 (±10)
**0.01% H_2_O_2_ (24 h)**	1.6	48.3	0.8	1.4	n.d.	n.d.
**0.1% H_2_O_2_ (24 h)**	5.8	97.4	2.7	3.7	n.d.	n.d.
**0.2% H_2_O_2_ (24 h)**	9.5	99.1	5.2	4.0	n.d.	n.d.
**0.3% H_2_O_2_ (24 h)**	86.9	100	11.5	8.3	n.d.	n.d.
**0.2% TBHP (24 h; n = 2)**	0.9 (±0.1)	73.2 (±0.7)	3.2 (±0.1)	5.0 (±0.2)	1.5 (±0.1)97.1 (±0.1)1.4 (±0.1)	96 (±10)
**0.7% TBHP (24 h; n = 4)**	2.0 (±0.6)	83.1 (±1.6)	6.3 (±0.1)	4.8 (±0.5)	4.5 (±0.1)92.5 (±0.5)3.0 (±0.4)	92 (±11)
**Control (7 d; n = 4))**	0.3 (±0.1)	2.4 (±0.4)	0.5 (±0.1)	1.0 (±0.3)	n.d.	n.d.
**0.05% TBHP (7 d; n = 6)**	0.5 (±0.2)	67.0 (±2.8)	13.3 (±1.3)	12.0 (±1.9)	0.6 (±0.1)96.8 (±0.1)2.6 (±0.1)	68 (±13)

Formation of fragments and aggregates was monitored by size-exclusion chromatography (Column 5) and target binding was assessed by SPR-analysis (Column 6). Data is represented as mean ± S.D; n.d. not determined.

The incubation of the selected IgG1 antibody with different H_2_O_2_ concentrations for 24 h resulted in increasing oxidation of Met-253, Met-83, and Trp-32 (Table 1). However, the significant oxidation of the buried Met-83 indicates degradation of the native antibody. In addition, significant formation of fragmentation products were observed by SDS-polyacrylamide gel electrophoresis under native conditions (data not shown). Since the structural integrity of the oxidized antibody is a prerequisite for the subsequent functional analysis of specific oxidized Met and Trp residues, the application of H_2_O_2_ was not further evaluated for the induction of Trp oxidation.

**Figure 4:**
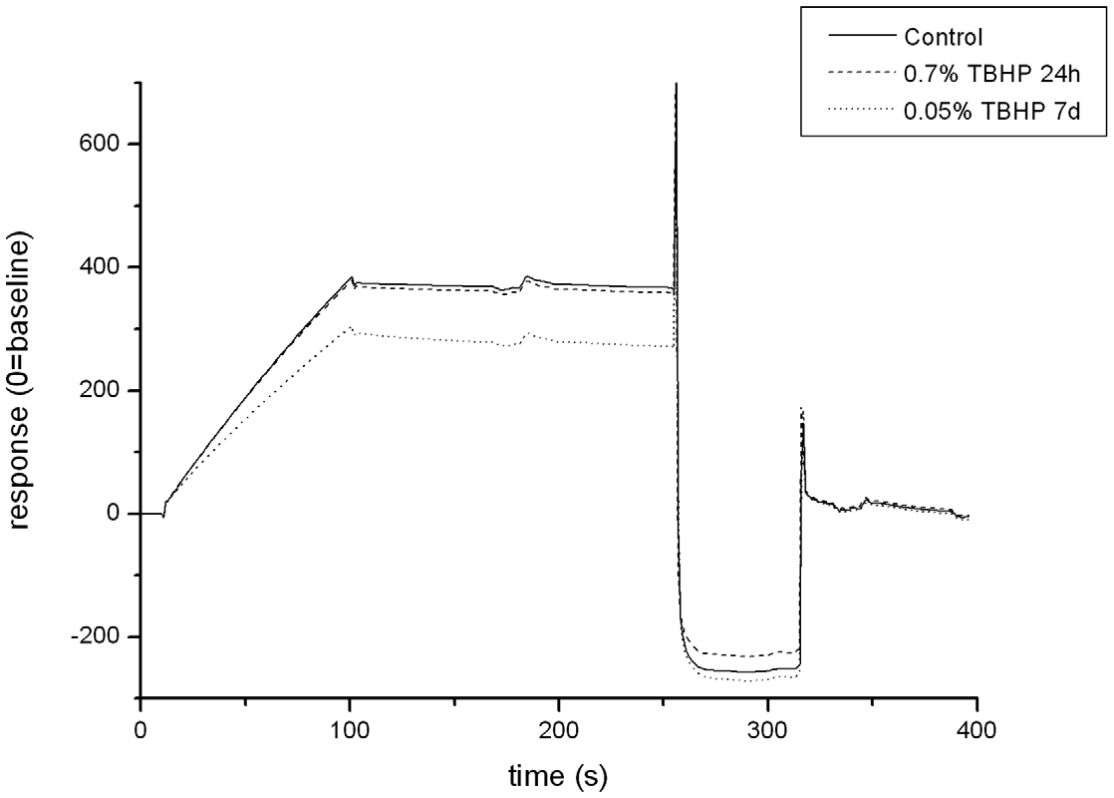
Analysis of antibody target binding by surface plasmon resonance. Biacore sensorgrams showing the pH 7.0 target binding (100 nM) following different TBHP exposures.

The incubation of the selected IgG1 antibody with different TBHP concentrations for 24 h resulted in increasing oxidation of Met-253 and Trp-32, respectively (Table 1). In addition, a slight increase in Met-83 oxidation was also observed at a concentration of 0.7% TBHP, albeit to a different extend when compared with the experiments applying H_2_O_2_ as oxidizing reagent (Table 1). The Trp-32 is located in the CDR1 region of the antibody light chain. Evaluation of antibody target binding activity by SPR-technology indicated a connection between the increase of Trp-32 oxidation and the loss of target binding activity (Table 1, Figure 4). However, the incubation of the selected IgG1 antibody with 0.7% TBHP also resulted in a significant formation of antibody fragments and aggregates when compared with the control study (Table 1). To minimize degradation events that might impact the functional analysis of the Trp-32 oxidation the TBHP concentration was lowered to 0.05% and the incubation time prolonged to 7 days.

These adaptations resulted in increasing oxidation of Trp-32 but did not alter the oxidation level of the buried Met-83 nor the formation of antibody fragments when compared with the control sample (incubation for 24 h w/o TBHP; Table 1). The formation of antibody aggregates was reduced when compared with the 0.7% TBHP incubation for 24 h and similar to the real-time stability study. Moreover, the increase of Trp-32 oxidation lead to a significant loss of target binding activity (Table 1, Figure 4). Thus, the achieved degree of Trp oxidation was adequate to identify the functional consequence of the Trp-32 oxidation. In this study, the Trp-32 of the selected IgG1 antibody was identified as being solvent accessible by x-ray crystallographic studies (data not shown). Thus, the facility of TBHP oxidation is governed by the solvent accessibility of the Met and Trp residues. Taken together, we have identified a single Trp residue in the CDR region of a recombinant IgG1 antibody susceptible to oxidation under real-time storage conditions and to TBHP oxidation.

## Discussion

Several studies have evaluated the oxidation of Met residues in recombinant antibodies using different TBHP concentrations for up to 24 h [3], [5], [6], [9], [10], [11]. However, significant formation of Trp oxidation has so far not been reported. Wei et al. identified a Trp residue in the CDR3 of the heavy chain that showed susceptibility to photooxidation but not for TBHP oxidation [6]. Shen et al. identified a Met residue (Met-107) in the CDR3 of the recombinant IgG1 antibody HER2 heavy chain that showed susceptibility to TBHP oxidation [9]. We analyzed the HER2 antibody after prolonged incubation (3 month) at elevated temperature conditions (35°C) and with the developed TBHP approach (0.05% TBHP for 7 days). We found strongly increased levels of Met-255 and moderately increased levels of Met-107 when compared with the HER2 reference material (Table 2). Comparing the Trp-32 oxidation level of the selected antibody with the Met-107 oxidation level of HER2, the extend of Trp-32 oxidation is significant. Taken together, the degree of light chain Trp-32 oxidation was found to be significantly higher than the oxidation levels of the conserved heavy chain Met-429 and the heavy chain Met-107 of the recombinant IgG1 antibody HER2, which have already been identified as being sensitive to chemical oxidation in previous studies. To review the presence of the Trp-32 residue in already published IgG1 antibody CDRL1 sequences a BLAST search using the patented protein database of GenBank was performed with the peptide sequence ASQGISSWLAWYQQK [19]. The database search resulted in the identification of >500 patented protein entries (with 100% sequence identity) underlining that Trp-32 is widely spread and of general interest for antibody development and stability testing.

**Table 2:** Relative quantification of HER2 methionine oxidation (Met-83, Met-107, and Met-255) by ion current chromatogram analysis of the oxidized product and its parent peptide using the quantification software GRAMS/32™.

	Met-83 (+16 Da)	Met-107 (+16 Da)	Met-255 (+16 Da)
**Reference material**	<0.2	<0.2	2.3
**Stability sample (3 M at 35**°**C)**	0.6	1.6	6.6
**Control (7 d; n = 2)**	<0.2	1.2 (±0.1)	2.5 (±0.8)
**0.05% TBHP (7 d; n = 3)**	<0.2	7.2 (±0.4)	72.3 (±0.7)

Oxidation is one of the major chemical degradation pathways for protein pharmaceuticals. Met in recombinant antibodies drew the greatest attention in several studies as it is most easily oxidized beside cysteine. To our knowledge, we have identified for the first time a solvent accessible Trp residue in the CDR region of a recombinant IgG1 antibody susceptible to oxidation under real-time storage and elevated temperature conditions. In order to reduce the time for simultaneous identification and functional evaluation of potential Met and Trp oxidation sites in the CDR of recombinant antibodies, a test system employing TBHP and quantitative LC-MS was developed. The developed model stress conditions allowed us to specifically oxidize the solvent accessible Met and Trp residues that displayed significant oxidation in the real-time stability and elevated temperature study but did not result in the oxidation of buried Met and Trp residues. The preservation of structural integrity was further demonstrated by SEC analysis where no fragment formation and only a minimal increase of aggregate formation was verified. Moreover, the achieved degree of Trp oxidation was adequate to identify the functional consequence of the Trp-32 oxidation by SPR analysis. In summary, the results obtained in this study demonstrate the high potential of employing TBHP combined with quantitative LC-MS with respect to identification and functional evaluation of Met and Trp oxidation sites in pharmaceutical research and development.

## Materials and Methods

### Preparation of oxidized MAb using *tert*-Butyl hydroperoxide or H_2_O_2_

To generate oxidized antibody, a recombinant IgG1 antibody was first diluted into 180 mM sodium chloride, 5 mM sodium acetate, pH 5.0 to a final concentration of 4 mg/mL. Subsequently, TBHP or H_2_O_2_ was added to the given percentage (v/v) and the samples were incubated for 24 h or 7 days at room temperature.

### Proteolytic digest of MAb

For detection and quantification of oxidized amino acids within the molecule, MAb was digested with trypsin. MAb was first denatured in 0.4 M Tris-HCl, 8 M Gua-HCl, at pH 8.5 by diluting 280 µg of oxidized MAb in a total volume of 300 µL. For reduction, 10 µl of 0.1 g/mL dithiothreitol (DTT) were added and incubated at 50°C for 1 hour. After alkylation of free cysteines by adding 0.33 g/mL iodoacetic acid (IAA) and incubation at room temperature in the dark for 30 min, the buffer was exchanged to digestion buffer (0.1 M Tris-HCl, pH 7.0) by application onto a NAP5^®^-gel filtration column (GE Healthcare, Buckinghamshire, UK). Subsequently, the NAP5^®^-eluate (500 µL) was mixed with 10 µL of a solution of 0.25 mg/mL trypsin (Trypsin Proteomics grade, Roche, Penzberg, Germany) in 10 mM HCl and incubated at 37°C for 18±2 h. The digest was stopped by adding 50 µl of a 10%-trifluoroacetic acid (TFA)-solution.

### Detection of oxidized peptides by liquid-chromatography mass-spectrometry (LC-MS)

The tryptic peptide mixture was separated by reversed phase-HPLC on a Jupiter C18 column (5 µm, 300 Å, 250×1 mm; Phenomenex, Aschaffenburg, Germany) and the eluate online analyzed with a Q-TOF 1 electrospray mass spectrometer (Waters, Manchester, UK). The mobile phases of RP-HPLC consisted of 0.1% TFA in water (solvent A) and 0.1% TFA in acetonitrile (solvent B). The chromatography was carried out using a step gradient from 0–25% solvent B in 33 min, from 25–40% solvent B in another 32 min and finally from 40–100% solvent B in 5 min. All experiments were performed on an Ultimate 3000 HPLC-system in µ-configuration (Dionex Softron GmbH, Germering, Germany) using a flow rate of 40 µL/min. The UV absorption was measured at a wavelength of 220 nm. 7 µg digested protein was applied. HPLC-system and mass spectrometer were connected by PEEK-capillary tubes. Before entering the mass spectrometer, a 3:1-mixture (v/v) of propionic acid and 2-propanol (TFA-fix) was added to the eluate to reduce the interference of TFA on the ionization of the peptides. Data acquisition was controlled by MassLynx™ software (Waters Micromass, Eschborn, Germany). Parameters for MS detection were adjusted according to general experience available from peptide analyses of recombinant antibodies.

### Data analysis for the quantification of oxidation levels

Peptides of interest were identified manually by searching their *m/z*-values within the experimental mass spectrum. For the quantification, specific ion current (SIC) chromatograms of peptides of interest were generated on the basis of their monoisotopic mass and detected charge states using GRAMS/32 software (Thermo Fisher Scientific, Dreieich, Germany). Relative amounts of non-oxidized and oxidized peptides were calculated by manual integration of the corresponding peaks.

### Identification of oxidized peptides by LC-MS/MS

MS/MS experiments were performed on-line on a LTQ Orbitrap mass spectrometer (Thermo Fisher Scientific) using the described chromatographic system. Peptides of interest were isolated on the basis of their mass and charge state and fragmentation induced by low-energy CID using helium as collision gas. The collision energy was adjusted according to stability and mass of the parent ion. Data acquisition was controlled by Xcalibur software (Thermo Fisher Scientific) using a manual acquisition mode. MS/MS-data were analyzed manually using Xcalibur software for mass detection and data interpretation.

### Size exclusion chromatography (SEC)

Size exclusion HPLC analysis of MAb was carried out using a TSK-Gel SW-3000 column (7.8×30 mm, 5 µm particle size; Tosoh Bioscience, Amsterdam, Netherlands). An isocratic elution using 200 mM KH_2_PO_4_, 250 mM potassium chloride, pH 7.0 at 0.5 mL/min as solvent was used for chromatographic separation on an Ultimate3000 HPLC-system equipped with UV detection at 280 nm. 150 µg of native or oxidized MAb was injected for the chromatographic analysis. SEC analyses were controlled and evaluated using Chromeleon software (Dionex Softron GmbH, Germering, Germany). Relative quantification of MAb and its variants was performed by manual integration of the chromatographic peaks and comparison of the respective areas.

### Analysis of target binding by surface plasmon resonance (SPR)

The interaction between the native or TBHP-treated MAb and the specific target receptor of MAb were measured by surface plasmon resonance using a Biacore T100 instrument (GE Healthcare Bioscience, Uppsala, Sweden). To evaluate MAb interaction to its specific target, the assay type “Calibration-free Concentration analysis” (BiaEvaluation Software, GE Healthcare Bioscience, Uppsala, Sweden) was performed. The extracellular domain of the receptor was immobilized onto a Biacore CM5-biosensor chip (GE Healthcare Bioscience, Uppsala, Sweden) via amine coupling to reach maximum coupling density. The assay was carried out at room temperature with HBS-P-buffer (GE Healthcare Bioscience) as running and dilution buffer. 5 nM of native or oxidized MAb samples were injected at a flow rate of 5 µL/min and 100 µL/min at room temperature, respectively. Association time was 90 s, dissociation phase took 60 s. Regeneration of the chip surface was reached by a short injection of 10 mM Glycine-HCl, pH 2.0. Evaluation of SPR-data was performed by comparison of the biological active amount of an untreated sample with TBHP-treated samples. Biological activity of untreated samples was fixed to 100% and oxidized MAb were set into relation.
